# Dexamethasone may improve severe COVID-19 via ameliorating endothelial injury and inflammation: A preliminary pilot study

**DOI:** 10.1371/journal.pone.0254167

**Published:** 2021-07-02

**Authors:** Won-Young Kim, Oh Joo Kweon, Min Jae Cha, Moon Seong Baek, Seong-Ho Choi

**Affiliations:** 1 Division of Critical Care Medicine, Department of Internal Medicine, Chung-Ang University Hospital, Chung-Ang University College of Medicine, Seoul, Republic of Korea; 2 Department of Laboratory Medicine, Chung-Ang University Hospital, Chung-Ang University College of Medicine, Seoul, Republic of Korea; 3 Department of Radiology, Chung-Ang University Hospital, Chung-Ang University College of Medicine, Seoul, Republic of Korea; 4 Division of Infectious Diseases, Department of Internal Medicine, Chung-Ang University Hospital, Chung-Ang University College of Medicine, Seoul, Republic of Korea; Georgetown Lombardi Comprehensive Cancer Center, UNITED STATES

## Abstract

Dexamethasone provides benefits in patients with coronavirus disease 2019 (COVID-19), although data regarding immunological profiles and viral clearance are limited. This study aimed to evaluate for differences in biomarkers among patients with severe COVID-19 who did and did not receive dexamethasone. We measured plasma biomarkers of lung epithelial/endothelial injury and inflammation in 31 patients with severe COVID-19 and in 13 controls. Changes in biomarkers and clinical parameters were compared during the 7-day period among COVID-19 patients, and also according to dexamethasone use. Thirty-two patients with severe COVID-19 who received mechanical ventilation (n = 6), high-flow nasal cannula (n = 11), and supplemental oxygen (n = 15) were analyzed. Relative to controls, patients with severe COVID-19 had significantly higher concentrations of biomarkers related to glycocalyx shedding (endocan and syndecan-1), endothelial injury (von Willebrand factor), and inflammation (soluble receptor for advanced glycation end-products [sRAGE] and interleukin-6). The 7-day decreases in biomarkers of endothelial injury (angiopoietin-2 [Ang-2] and intercellular adhesion molecule-1 [ICAM-1]) and sRAGE, but not in the biomarker of lung epithelial injury (surfactant protein D), were correlated with decreases in C-reactive protein and radiologic score at day 7. Twenty patients (63%) received dexamethasone, and the dexamethasone and non-dexamethasone groups differed in terms of disease severity. However, dexamethasone was associated marginally with increased SpO_2_/FiO_2_ and significantly with decreases in C-reactive protein and radiologic score after adjusting for baseline imbalances. Furthermore, the dexamethasone group exhibited a significant decrease in the concentrations of Ang-2, ICAM-1, soluble form of the Tie2 receptor (a biomarker of glycocalyx shedding), and sRAGE. Both groups exhibited a clinically insignificant increase in the cycle threshold value. Severe COVID-19 may be characterized by more severe endothelial injury and inflammation, and less severe lung epithelial injury. There is a possibility that dexamethasone improved severe COVID-19 and related endothelial injury without delaying viral clearance.

## Introduction

As of February 10, 2021, there were >106 million cases of coronavirus disease 2019 (COVID-19) in 223 countries, which had resulted in nearly 2,320,000 confirmed deaths [1]. Most patients experience mild to moderate disease, although 5–10% of cases progress to severe or critical disease, which can involve pneumonia and acute respiratory distress syndrome (ARDS) [2]. Severe COVID-19 cases typically involve a mild-to-moderate presentation followed by secondary respiratory failure at 9–12 days after the first onset of symptoms [2, 3]. The concept of cytokine storm syndrome emerged early in the pandemic to explain critically ill patients with COVID-19 [4]. However, accumulating evidence challenges the single cytokine storm model and suggests that severe COVID-19 is a dysregulated host response of inflammation, immunity, and interferon signaling [5, 6].

Dexamethasone is currently the only agent that can provide a survival benefit in COVID-19 cases [7]. This survival benefit was greater for patients who were receiving supplemental oxygen or mechanical ventilation and was not observed among patients who were not receiving oxygen. There are questions regarding the benefits of dexamethasone for patients with severe COVID-19, given the controversy regarding corticosteroid treatment of non-COVID-19 ARDS and other viral pneumonias [8]. Thus, it would be useful to collect data regarding immunological features and viral clearance from before and after dexamethasone, although the previous trials of corticosteroid treatment for COVID-19 did not collect these data [7, 9–11]. Therefore, the present study aimed to evaluate for differences in biomarkers among patients with severe COVID-19 who did and did not receive dexamethasone. In addition, this study evaluated whether dexamethasone could impair viral clearance or antiviral immunity.

## Materials and methods

### Study design, subjects, and setting

This study evaluated adult patients with COVID-19 who were hospitalized in an 835-bed university-affiliated tertiary hospital in Seoul, Korea between February 2020 and November 2020. The study protocol was approved by the institutional review board of Chung-Ang University Hospital (2092-001-432). The confirmation of COVID-19 was based on upper respiratory tract specimens that were positive for severe acute respiratory syndrome coronavirus 2 (SARS-CoV-2) according to a real-time reverse transcription polymerase chain reaction (rRT-PCR) assay [12]. The present study included patients with severe COVID-19 who received mechanical ventilation, high-flow nasal cannula, or supplemental oxygen, and excluded COVID-19 patients who did not receive oxygen. Thirteen healthy individuals who had voluntarily undergone a private health examination at the same hospital during November 2020 were recruited as controls. To minimize confounding factors related to the patients’ clinical characteristics, the cases and controls were randomly matched according to age and sex.

The patients with COVID-19 who were admitted to our hospital between February–June 2020 were not treated with corticosteroids because of the weak recommendations from various guidelines [13, 14] and thus formed the non-dexamethasone group. Based on a press release regarding the results of the RECOVERY trial (June 16, 2020), our hospital subsequently adopted dexamethasone, which was administered orally or intravenously according to the RECOVERY protocol (6 mg every 24 h for up to 10 days) [7], as routine adjuvant therapy for severe COVID-19. Thus, the patients with severe COVID-19 who were admitted between June–November 2020 formed the dexamethasone group. Baseline demographics, clinical outcomes, physiological characteristics, and plasma biomarkers were compared between the groups. During the first wave of the epidemic in Korea (February–March 2020), the city of Seoul had a relatively low number of confirmed cases. However, an increased number of patients required high-flow nasal cannula and mechanical ventilation at our hospital during the second wave of the epidemic (August 2020). Therefore, there were clear imbalances between the dexamethasone non-dexamethasone groups in terms of disease severity.

### Biomarker assays

To investigate clinical characteristics and viral shedding of COVID-19, our institution had prospectively collected paired blood and saliva samples at several time-points from the day of admission to hospital discharge in patients with COVID-19. All samples had been collected after receiving informed written consent from the patient or their next of kin. For the present study, we measured plasma biomarkers of lung epithelial/endothelial injury and inflammation using these samples.

Few studies have identified diagnostic and prognostic biomarkers in cases of severe COVID-19. Thus, we selected a panel of biomarkers that have performed well in the diagnosis of non-COVD-19 ARDS [15, 16]. These biomarkers include angiopoietin-2 (Ang-2), intercellular adhesion molecule-1 (ICAM-1), soluble receptor for advanced glycation end-products (sRAGE), surfactant protein D (SP-D), and von Willebrand factor (vWF). Pathological shedding of transmembrane proteins involved in the glycocalyx structure was evaluated based on the soluble form of the Tie2 receptor (sTie2), endocan, and syndecan-1. Furthermore, plasma concentrations of interferon-α (IFN-α) and viral loads in the upper and/or lower respiratory tract specimens were compared between the dexamethasone and non-dexamethasone groups, as type I IFNs are essential for antiviral immunity and glucocorticoids can impair IFN-mediated viral clearance [17]. Finally, traditional markers of inflammation were measured, such as interleukin-6 (IL-6) and tumor necrosis factor-α (TNF-α).

With the exception of IFN-α, all other biomarkers in the plasma samples were measured using the Luminex^®^ Assay Human Premixed Multi-Analyte Kit (R&D Systems, Minneapolis, MN, USA). Plasma concentrations of IFN-α were measured using an enzyme-linked immunosorbent assay kit (Human IFN-α ELISA Kit; Novus Biologicals, Centennial, CO, USA). All assays were performed according to the manufacturer’s instructions.

### Data collection and definitions

Day 1 was defined as the first day of dexamethasone for the dexamethasone group and as the first day of supplemental oxygen or mechanical ventilation for the non-dexamethasone group. Baseline data were collected regarding age, sex, body mass index (BMI), smoking status, Charlson Comorbidity Index [18], date of symptom onset, ARDS, quick Sequential Organ Failure Assessment score [19], and the type of oxygen support on day 1. Moreover, data were extracted on days 1, 4, and 7 regarding vital signs, laboratory findings, radiologic findings, rRT-PCR results, and biomarker assay results. The consensus definition was used to identify ARDS [20]. Hypoxemia was evaluated based on SpO_2_/FiO_2_, instead of PaO_2_/FiO_2_, as arterial blood gas data were not available for every patient [21]. Anteroposterior chest radiography results were reviewed by a thoracic radiologist (M.J.C.) to calculate chest radiographic scores as previously described [22]. The cycle threshold (Ct) values from the rRT-PCR tests reflect the threshold for identifying viral amplicons and are inversely correlated with the amount of RNA that is present. Thus, a lower Ct value indicates a relatively large amount of viral RNA. The number of oxygen-free days at day 28 was defined as the number of days that the patient was alive and free from any oxygen support for ≥48 h. Patients who were discharged alive from the hospital before 28 days were considered free from oxygen support at 28 days. Superinfection was diagnosed if a new microbiological infection emerged at ≥48 h after admission.

### Statistical analysis

Continuous variables were presented as median (interquartile range, IQR) or as mean ± standard deviation, and were compared using the Mann-Whitney *U* test. Categorical variables were presented as number (percentage) and were compared using the chi-squared test or Fischer’s exact test, as appropriate. Logistic regression models were used to calculate odds ratios (ORs) and 95% confidence interval (CIs) to determine the ability of different biomarkers to predict severe COVID-19. Identification of an optimal cut-off value for each variable of interest was based on Youden’s index [23]. Multivariate logistic regression analysis was performed to adjust for age, sex, BMI, diabetes, hypertension, and serum creatinine, all of which may affect the levels on some biomarkers [24]. Changes in plasma biomarkers and clinical parameters at day 4 and day 7 were evaluated using the two-tailed Spearman correlation coefficient (non-parametric). To avoid multiple comparisons, the areas under the curve (AUCs, relative to the baseline values) were calculated for continuous variables with repeated measurements and were compared between the dexamethasone and non-dexamethasone groups using the Mann Whitney *U* test, as previously described [25] (S1 Methods). The AUC values were calculated relative to the baseline values in order to account for baseline imbalances between the two groups. All tests were two-tailed, and differences were considered statistically significant at *P*-values of <0.05. All analyses were performed using IBM SPSS software (version 26.0; IBM Corp., Armonk, NY, USA) and MedCalc for Windows (version 19.8; MedCalc Software, Ostend, Belgium).

## Results

### Patient characteristics

During the study period, 62 consecutive patients with COVID-19 were admitted to our hospital, although 30 patients were excluded because they did not receive oxygen treatment. The remaining 32 patients were included in the analysis, and their baseline characteristics and clinical outcomes are described in Table 1. The median time from symptom onset to day 1 was 8 days (IQR: 6–11 days). All patients had pneumonia and the prevalence of ARDS was 22% (7/32 patients). The in-hospital oxygen support included mechanical ventilation (6 patients, 19%), high-flow nasal cannula (11 patients, 34%), and supplemental oxygen (15 patients, 47%). Eleven patients (34%) received remdesivir after July 2, 2020, and 20 patients (63%) received dexamethasone after June 30, 2020.

**Table 1:** Baseline characteristics and clinical outcomes of the 32 patients with severe COVID-19.

Baseline characteristics	
Age, years	70 (60–76)
Male sex	17 (53)
Body mass index, kg/m^2^	24.4 (22.0–27.8)
Smoking status	
Ever smoker	14 (44)
Never smoker	18 (56)
Comorbidities	
Diabetes	8 (25)
Hypertension	18 (56)
Chronic lung disease	7 (22)
Liver cirrhosis	1 (3)
Malignancy	1 (3)
Charlson Comorbidity Index	3.0 (2.0–4.0)
Time from symptom onset to day 1, days	8 (6–11)
ARDS	7 (22)
qSOFA score	1 (0–1)
Type of oxygen support	
Mechanical ventilation	6 (19)
High-flow nasal cannula	11 (34)
Supplemental oxygen	15 (47)
Remdesivir	11 (34)
Dexamethasone	20 (63)
Duration, days	10 (9–13)
Vital signs and laboratory data	
Body temperature, °C	37.9 (37.3–38.3)
Mean blood pressure, mmHg	82 (73–91)
Heart rate, beats/min	86 (77–94)
Respiratory rate, breaths/min	20 (20–24)
SpO_2_/FiO_2_	248 (192–348)
Creatinine, mg/dL	0.7 (0.6–0.8)
White cell count, 1000/mm^3^	6.0 (3.8–7.8)
Total bilirubin, mg/dL	0.7 (0.5–0.8)
Lactate dehydrogenase, IU/L	395 (286–445)
C-reactive protein, mg/L	78 (41–148)
Glucose, mg/dL	126 (109–156)
Radiologic score	4 (2–6)
Cycle threshold value	25.4 (20.5–29.0)
**Clinical outcomes**	
Oxygen-free days at day 28	13.4 ± 8.9
Length of hospital stay, days	19.8 ± 13.6
Hospital mortality	1 (3)
Superinfection	2 (6)

Data are presented as number (%), mean ± standard deviation, or median (interquartile range).

ARDS, acute respiratory distress syndrome; COVID-19, coronavirus disease 2019; FiO_2_, fraction of inspired oxygen; SpO_2_, pulse oximetric saturation; qSOFA, quick Sequential Organ Failure Assessment.

The in-hospital mortality rate was 3% (1/32 patients). The mean number of oxygen-free survival days during the first 28 days was 13.4 ± 8.9 days. Two patients (6%) who received dexamethasone had superinfection during hospitalization, which included one case of a catheter-associated urinary tract infection and one case of ventilator-associated pneumonia.

### Plasma biomarkers

**Fig 1:**
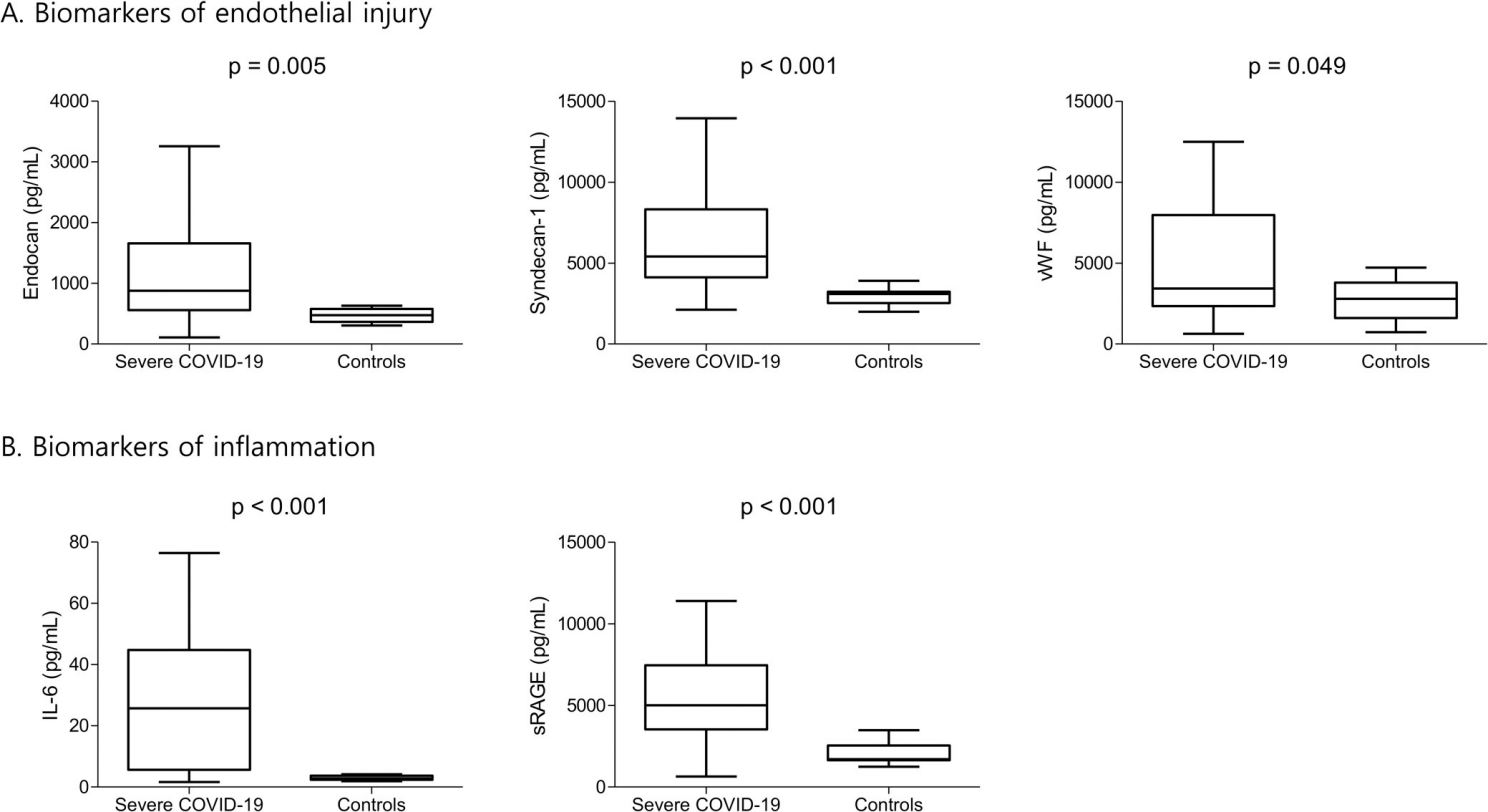
Plasma biomarkers of endothelial injury and inflammation in patients with severe COVID-19 and healthy individuals. Center lines represent the median values, the box tops and bottoms represent the interquartile ranges, error bars represent the overall ranges. COVID-19, coronavirus disease 2019; IL-6, interleukin-6; sRAGE, soluble receptor for advanced glycation end-products; vWF, von Willebrand factor.

Table 2 and Fig 1 show the biomarker concentrations for patients with severe COVID-19 and the healthy individuals. One patient with severe COVID-19 was excluded from the analysis because of refusal to participate. Relative to the controls, the patients with severe COVID-19 had significantly higher concentrations of biomarkers for glycocalyx shedding (endocan and syndecan-1) and endothelial injury (vWF). Furthermore, the patients with severe COVID-19 had significantly higher concentrations of sRAGE and IL-6 (biomarkers of inflammation). Moreover, the patients with severe COVID-19 had a significantly increased type I IFN response, which is essential for antiviral immunity. However, there were no significant inter-group differences in terms of the plasma concentrations of Ang-2, sTie2, ICAM-1, SP-D, and TNF-α. These findings were generally consistent when we evaluated patients with hypertension (S1 Table).

**Table 2:** Comparing plasma biomarker concentrations between 31 patients with severe COVID-19 and 13 healthy individuals.

	Severe COVID-19 (n = 31)	Controls (n = 13)	*P*
Ang-2, pg/mL	1313 (849–1966)	1132 (1002–1248)	0.827
sTie2, pg/mL	16193 (12974–20237)	21198 (17614–23434)	0.066
Endocan, pg/mL	878 (616–1618)	477 (368–548)	0.005
ICAM-1, pg/mL	428844 (311242–625396)	366880 (293338–671385)	0.787
IL-6, pg/mL	25.7 (8.8–43.9)	2.8 (2.4–3.7)	<0.001
sRAGE, pg/mL	5004 (3557–7280)	1706 (1656–2536)	<0.001
SP-D, pg/mL	6234 (3038–10756)	6381 (5112–7067)	0.969
Syndecan-1, pg/mL	5401 (4208–7734)	3091 (2572–3233)	<0.001
TNF-α, pg/mL	7.9 (6.1–9.0)	7.0 (6.0–8.0)	0.432
vWF, pg/mL	3436 (2356–7829)	2793 (2079–3517)	0.049
IFN-α, pg/mL	129.3 (41.6–183.8)	16.1 (13.8–18.2)	<0.001

Data are presented as median (interquartile range) and *P*-values were calculated using the Mann-Whitney *U* test.

Ang-2, angiopoietin-2; COVID-19, coronavirus disease 2019; ICAM-1, intercellular adhesion molecule-1; IFN-α, interferon-α; IL-6, interleukin-6; sTie2, soluble form of the Tie2 receptor; sRAGE, soluble receptor for advanced glycation end-products; SP-D, surfactant protein D; TNF-α, tumor necrosis factor-α; vWF, von Willebrand factor.

Multivariate analysis, which was adjusted for age, sex, BMI, diabetes, hypertension, and serum creatinine, revealed that endocan ≥632.25 pg/mL (adjusted OR: 293.42, 95% CI: 5.65–15242.31; p = 0.005), IL-6 ≥4.19 pg/mL (adjusted OR: 26.13, 95% CI: 4.10–166.60; p = 0.001), sRAGE ≥3487.12 pg/mL (adjusted OR: 15.58, 95% CI: 2.65–91.57; p = 0.002), and syndecan-1 ≥3908.58 pg/mL (adjusted OR: 43.20, 95% CI: 4.47–417.27; p = 0.001) were associated with severe COVID-19 (Table 3).

**Table 3:** Multivariate logistic regression analysis using plasma biomarkers to predict severe COVID-19.

	Unadjusted OR (95% CI)	*P*	Adjusted OR^a^ (95% CI)	*P*
Ang-2 ≥1247.91 pg/mL	2.40 (0.61–9.47)	0.211	2.87 (0.53–15.49)	0.220
Endocan ≥632.25 pg/mL	34.50 (3.85–309.16)	0.002	293.42 (5.65–15242.31)	0.005
ICAM-1 ≥213785.49 pg/mL	2.64 (0.33–21.09)	0.361	1.87 (0.16–21.40)	0.613
IL-6 ≥4.19 pg/mL	22.92 (3.98–131.93)	<0.001	26.13 (4.10–166.60)	0.001
sRAGE ≥3487.12 pg/mL	18.86 (3.36–105.93)	0.001	15.58 (2.65–91.57)	0.002
Syndecan-1 ≥3908.58 pg/mL	50.00 (5.40–463.18)	0.001	43.20 (4.47–417.27)	0.001
TNF-α ≥8.49 pg/mL	2.11 (0.48–9.24)	0.324	2.52 (0.36–17.67)	0.352
vWF ≥4724.52 pg/mL	8.67 (0.998–75.24)	0.050	4.37 (0.43–44.18)	0.212

Ang-2, angiopoietin-2; CI, confidence interval; COVID-19, coronavirus disease 2019; ICAM-1, intercellular adhesion molecule-1; IL-6, interleukin-6; OR, odds ratio; sRAGE, soluble receptor for advanced glycation end-products; TNF-α, tumor necrosis factor-α; vWF, von Willebrand factor.

^a^ Adjusted for age, sex, body mass index, diabetes, hypertension, and serum creatinine.

**Fig 2:**
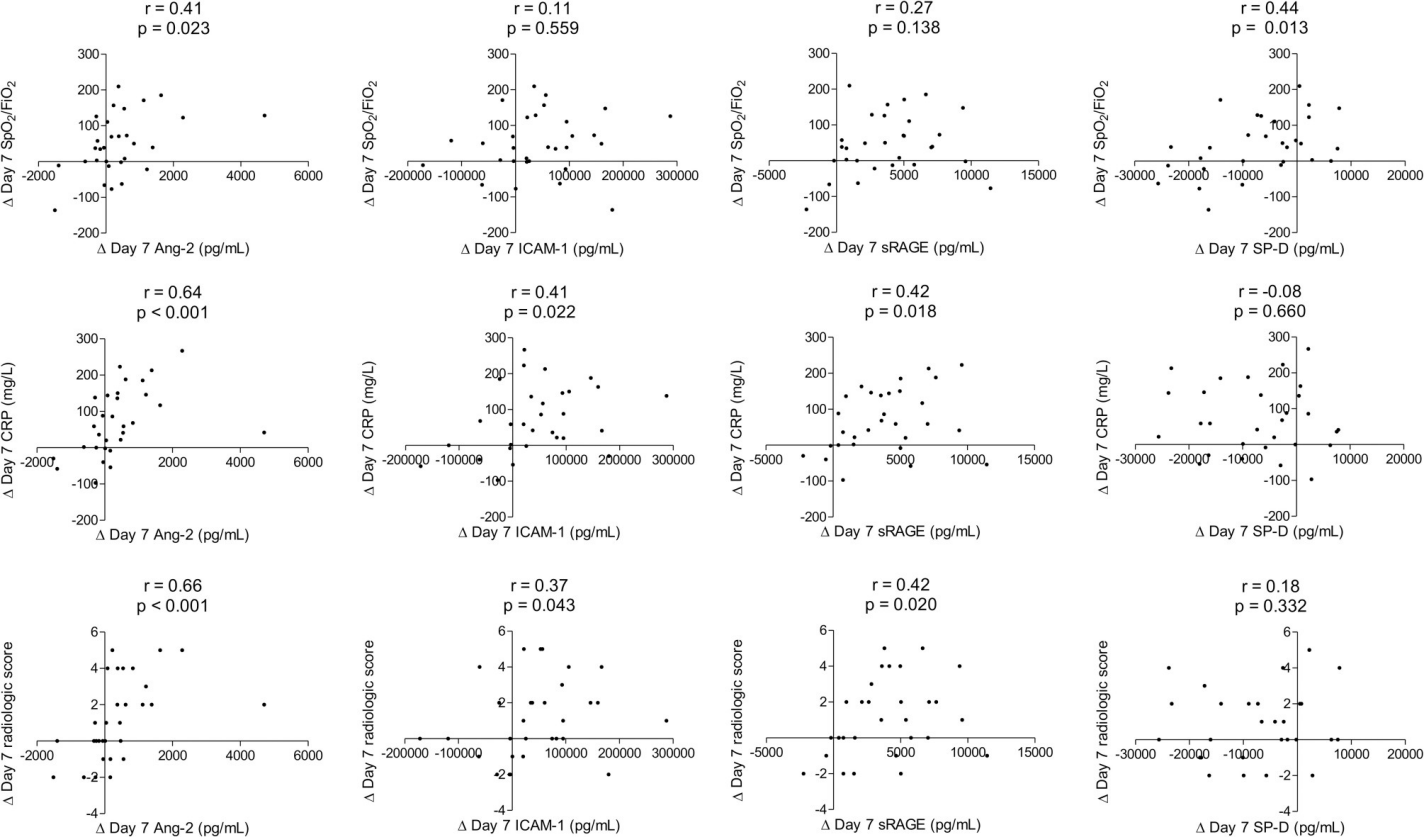
Changes in plasma biomarkers and clinical parameters over the 7-day study period. Ang-2, angiopoietin-2; CRP, C-reactive protein; FiO_2_, fraction of inspired oxygen; ICAM-1, intercellular adhesion molecule-1; SpO_2_, pulse oximetric saturation; sRAGE, soluble receptor for advanced glycation end-products; SP-D, surfactant protein D.

The changes in plasma biomarkers were evaluated for associations with improvements in clinical parameters. The 7-day decrease in Ang-2 concentration (a biomarker of endothelial injury) was correlated with 7-day increase in SpO_2_/FiO_2_ and decreases in C-reactive protein (CRP) and radiologic score (Fig 2 and S2 Table). In addition, the 7-day decreases in ICAM-1 concentration (another biomarker of endothelial injury) and sRAGE concentration were correlated with 7-day decreases in CRP and radiologic score. However, the 7-day decrease in SP-D concentration (a biomarker of lung epithelial injury) was only correlated with a 7-day increase in SpO_2_/FiO_2_. Analysis of the correlations between changes in other plasma biomarkers and clinical parameters at day 4 and day 7 are described (S2 Table).

### Characteristics of the dexamethasone and non-dexamethasone groups

The patients’ baseline characteristics and clinical outcomes according to dexamethasone use are described (S3 Table). There were no significant inter-group differences in terms of the median time from symptom onset to day 1 or the proportion of patients with ARDS. As expected, the dexamethasone group had greater use of mechanical ventilation and high-flow nasal cannula, significantly lower SpO_2_/FiO_2_ ratios, significantly higher CRP concentrations, and significantly higher radiologic scores. None of the patients in the non-dexamethasone group received any type of corticosteroid treatment. Furthermore, the patients in the non-dexamethasone group did not receive remdesivir, as this drug was only distributed in Korea after July 2, 2020. Nevertheless, comparisons of the two groups revealed similar values for viral load, in-hospital mortality rate, and oxygen-free days at day 28.

### Clinical parameters and plasma biomarkers according to dexamethasone use

**Fig 3:**
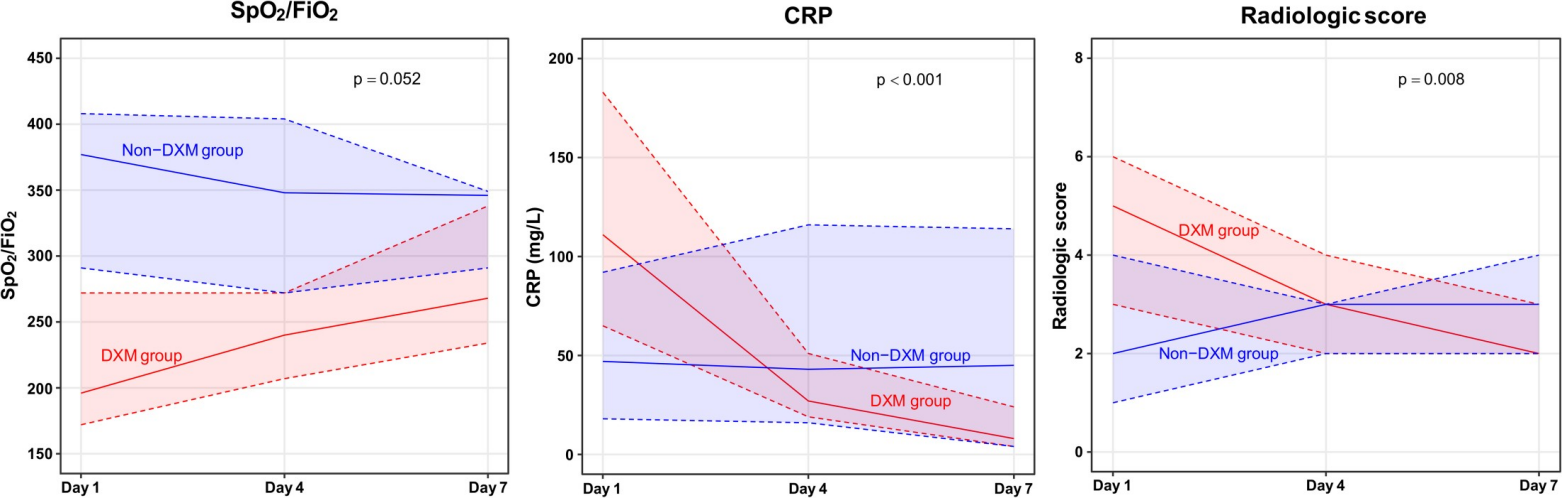
Clinical parameters during the 7-day study period. Solid lines represent the median values and the shaded areas bordered by dotted lines represent the upper and lower quartiles. CRP, C-reactive protein; DXM, dexamethasone; FiO_2_, fraction of inspired oxygen; SpO_2_, pulse oximetric saturation.

The 7-day changes in clinical parameters are shown in Fig 3 and S4 Table. The dexamethasone group tended to have a greater increase in SpO_2_/FiO_2_ based on the median AUC values (40 [IQR: -3 to 70] vs. -2 [IQR: -32 to 28]; p = 0.052). Furthermore, the dexamethasone group had a markedly decreased CRP concentration (median AUC: -58 mg/L [IQR: -113 to -30 mg/L] vs. 10 mg/L [IQR: 0 to 30 mg/L]; p < 0.001) and a significantly decreased radiologic score (median AUC: -1 [IQR: -3 to 0] vs. 0 [IQR: 0 to 1]; p = 0.008).

**Fig 4:**
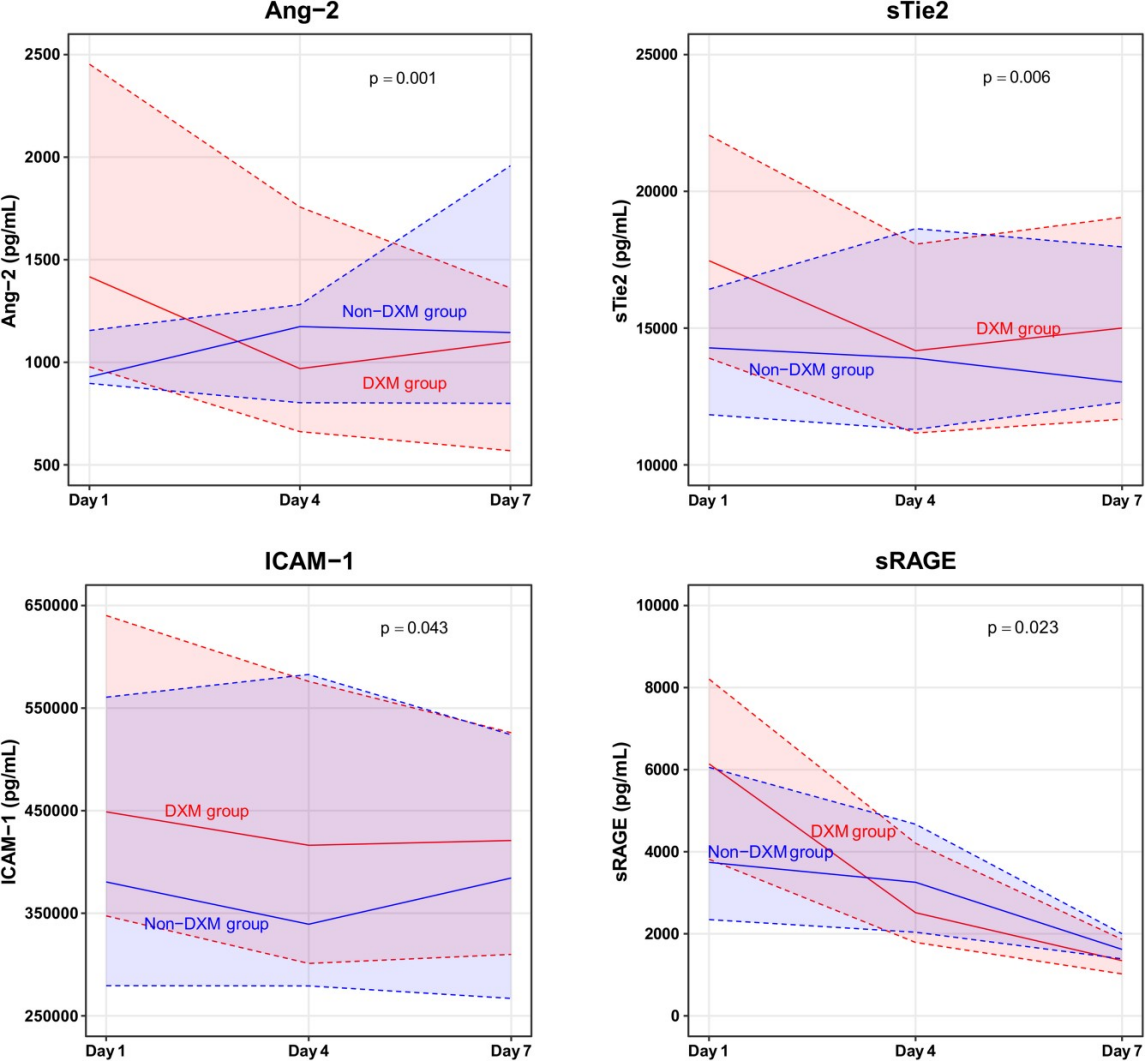
Plasma biomarkers during the 7-day study period. Solid lines represent the median values and the shaded areas bordered by dotted lines represent the upper and lower quartiles. Ang-2, angiopoietin-2; DXM, dexamethasone; ICAM-1, intercellular adhesion molecule-1; sTie2, soluble form of the Tie2 receptor; sRAGE, soluble receptor for advanced glycation end products.

The 7-day changes in plasma biomarkers are shown in Fig 4 and S4 Table. The dexamethasone group exhibited significant decreases in biomarkers of endothelial injury (Ang-2 and ICAM-1), glycocalyx shedding (sTie2), and inflammation (sRAGE). Serial analyses of the other clinical parameters and plasma biomarkers at days 1, 4, and 7 are described (S4 Table).

**Fig 5:**
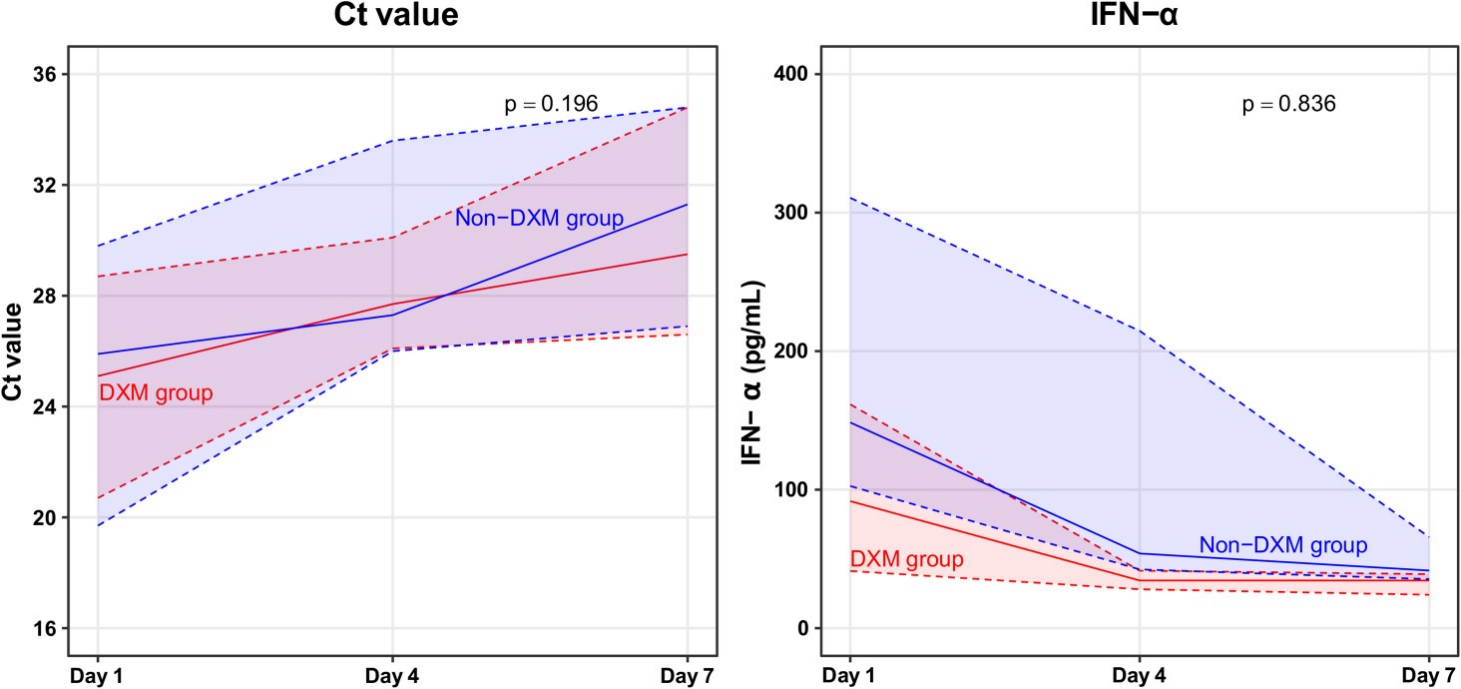
Severe acute respiratory syndrome coronavirus 2 load and type I interferon response during the 7-day study period. Solid lines represent the median values and the shaded areas bordered by dotted lines represent the upper and lower quartiles. Ct, cycle threshold; DXM, dexamethasone; IFN-α, interferon-α.

The SARS-CoV-2 loads and type I IFN responses were compared according to dexamethasone. During the 7-day study period, the dexamethasone and non-dexamethasone groups exhibited increases in their Ct values, although the difference was not clinically relevant (median AUC: 3.9 [IQR: 2.1 to 4.8] vs. 5.3 [IQR: 3.7 to 5.8]; p = 0.196) (Fig 5 and S4 Table). The median IFN-α concentration at day 1 was non-significantly lower in the dexamethasone group (91.7 pg/mL [IQR: 41.3–161.6 pg/mL] vs. 148.5 pg/mL [IQR: 102.6–310.7 pg/mL]; p = 0.302). However, both groups exhibited 7-day decreases in the IFN-α concentration, and the difference was not clinically relevant (p = 0.836).

## Discussion

The present study revealed four main findings. First, severe COVID-19 may be characterized by more severe endothelial injury and inflammation, relative to lung epithelial injury. Second, a decrease in the levels of biomarkers of endothelial injury was associated with clinical improvement. Third, dexamethasone might have improved the clinical course of severe COVID-19 and related endothelial injury. Fourth, dexamethasone did not seem to delay viral clearance.

Unlike with other respiratory viruses, viral shedding in SARS-CoV-2 appears to be increased early in the illness [26]. After the first week of illness, early studies noted substantially elevated pro-inflammatory cytokine concentrations in patients with COVID-19 who required critical care [4]. However, other investigators noted modestly elevated IL-6 concentrations with high rates of thrombosis and markedly elevated D-dimer concentrations, which suggested that COVID-19 involved hypo-inflammatory vasculopathy rather than an inflammatory cytokine storm [27, 28]. To further clarify the mechanisms of lung and systemic injury in COVID-19, we tested 10 biomarkers of endothelial injury, glycocalyx shedding, inflammation, and lung epithelial injury over the first 7 days of treatment for patients with severe COVID-19. We unexpectedly observed that, relative to healthy control subjects, patients with severe COVID-19 did not have significantly elevated markers of endothelial injury, such as Ang-2 and ICAM-1. However, severe COVID-19 was associated with marked increases in endocan and syndecan-1, which reflect pathological shedding of glycocalyx. These findings are consistent with a previous report that endothelial glycocalyx shedding may predict widespread endothelial injury in severe COVID-19 [29].

Type II alveolar cells produce SP-D, which is a normal constituent of pulmonary surfactant. Elevated SP-D concentrations have been reported in patients with non-COVID-19 ARDS [15, 16]. However, the present study did not identify significantly increased SP-D concentrations in the patients with COVID-19, relative to in the healthy controls. In addition, decreased Ang-2 and ICAM-1 concentrations at day 7 were associated with decreases in CRP concentration and radiologic score at day 7, but changes in SP-D concentration were not. Thus, although the virus initially affects the airway epithelial cells, the airway epithelium may not be destroyed for a considerable time. Instead, the increased viral load appears to lead to viremia, endothelialitis, and potentially multiorgan failure. Therefore, our findings suggest that biomarkers of endothelial injury and glycocalyx degradation may be the most reliable indicators for diagnosis and prognostication in cases of severe COVID-19. Interestingly, although SP-D was not associated with severe COVID-19, we did detect a significantly increased concentration of sRAGE in these patients. Furthermore, a decreased sRAGE concentration at day 7 was associated with decreases in CRP concentration and radiologic score at day 7. Type I alveolar cells highly express sRAGE, which has been used as a marker of lung epithelial injury [30, 31], although sRAGE also plays an important role in the innate immune response [32]. The lack of a significant relationship with SP-D may be related to the pro-inflammatory role of sRAGE in severe COVID-19. Our findings of sRAGE are further supported by a recent study demonstrating that the levels of total sRAGE were upregulated in diabetic and non-diabetic COVID-19 patients [33]. In contrast, Yalcin Kehribar et al. observed high sRAGE levels in young patients with asymptomatic COVID-19 and low sRAGE levels in elderly patients with lung involvement [34]. One possible explanation for this discrepancy is that sRAGE levels may help predict early mortality but appear to be less helpful for patients already having severe disease. For instance, sRAGE was a good predictor of the need for mechanical ventilation as well as mortality among patients with COVID-19 [35], although RAGE levels one day after mechanical ventilation did not differ between survivors and non-survivors with COVID-19 ARDS [36]. RAGE signaling is also upregulated in chronic inflammation including cardiovascular diseases [37], although our finding that the patients with severe COVID-19 had significantly higher concentrations of sRAGE than the healthy controls were not influenced among patients with hypertension.

Preclinical and clinical studies of ARDS have shown that corticosteroid treatment rescues the cellular concentrations and functions of glucocorticoid receptors, which can lead to biological improvement and disease resolution [38]. In patients with severe COVID-19, glucocorticoid receptor expression in bronchoalveolar lavage myeloid cells is inversely related to lung inflammation and symptom severity [39]. Although the results from randomized trials [7, 10, 11] and a meta-analysis [40] confirmed the beneficial effects of corticosteroids for critically ill patients with COVID-19, they did not consider laboratory parameters regarding oxygenation and inflammation, radiologic findings, and biomarkers. In our study, even after adjusting for baseline imbalances, dexamethasone was associated with increased SpO_2_/FiO_2_, decreased CRP concentrations, and decreased radiologic scores. Moreover, markers of endothelial injury and inflammation decreased after dexamethasone administration. Additional data are needed to evaluate whether corticosteroid dose, duration, and dose tapering could be guided by these findings.

In mild COVID-19 cases, retrospective studies suggest that corticosteroid use may be associated with prolonged viral shedding [41]. The present study did not detect delayed viral clearance in patients with severe COVID-19 who received dexamethasone, although it is possible that viral replication had already declined in most patients, given that SARS-CoV-2 may be largely eradicated within 7 days in most patients [7]. In this context, type I IFNs have direct antiviral effects via inhibition of viral replication [42]. In addition, attenuation of the type I IFN response induces various inflammatory cytokines and dampens an effective T-cell response [43], while the SARS-CoV-2 genome encodes proteins that antagonize type I IFNs [44]. A recent study of patients with severe and critical COVID-19 identified undetectable IFN-β concentrations, low IFN-α production and activity, and persistent plasma viral loads [5]. Similarly, relative to the non-dexamethasone group, our dexamethasone group had more severe disease and lower baseline concentrations of IFN-α. Corticosteroid treatment during the early phase of viral infection might suppress the host antiviral responses by impairing IFN-mediated viral clearance [17]. We observed that IFN-α concentrations in the dexamethasone group continued to decrease during the study period, although a similar pattern was observed in the non-dexamethasone group, which makes it difficult to interpret the potential association of corticosteroid use with a reduced antiviral response.

To the best of our knowledge, ours is the first comprehensive study to test a multi-pathway panel of biomarkers in patients with severe COVID-19. Relative to cross-sectional studies that only considered single biomarkers, our findings regarding serial changes in 10 biomarkers and clinical parameters provide important new information regarding the pathophysiology of severe COVID-19. Our results may help guide the management of patients with severe COVID-19 and also help explain how corticosteroids provide a benefit in these patients. We also evaluated the effects of dexamethasone on viral load and antiviral immunity. Thus, despite the small sample size, our findings may help improve our understanding of the mechanisms underlying severe COVID-19.

This study has several limitations that must be considered. First, the single-center retrospective design is associated with various risks of bias, and the small sample size might have limited the power of the analyses and could explain some non-significant results. We calculated the AUCs relative to the baseline values to control for baseline differences between the groups, but multiple confounding factors still exist. Thus, randomized trials would be needed to establish causality, although it would be unethical to conduct another corticosteroid vs. placebo trial due to updated guidelines recommending the use of corticosteroids in severe COVID-19. Second, we only considered 10 biomarkers, which is only a subset of all potential biomarkers for diagnosis and prognostication in cases of severe COVID-19. Moreover, we did not assess the immune responses within the lungs. Third, we measured the viral load using upper and/or lower respiratory tract specimens, instead of blood, which might not be a true surrogate marker of systemic infection. Fourth, patients in the non-dexamethasone group did not receive remdesivir, which might be an important treatment for COVID-19. Fifth, the radiologic score can depend on the quality of the radiographic images and observer experience, although our thoracic radiologist has 6 years of experience and previous studies have indicated that the radiologic score has excellent inter-observer agreement [22]. Finally, we selected patients who were most likely to benefit from dexamethasone, and the results should not be extrapolated to patients who do not receive oxygen therapy.

## Conclusions

In conclusion, severe COVID-19 may be characterized by more severe endothelial injury and inflammation, relative to lung epithelial injury. Dexamethasone may provide a greater benefit in terms of managing endothelial injury. However, independent validation in a larger sample is needed to address the present study’s findings.

## Supporting information

S1 Table Comparing plasma biomarker concentrations between patients with hypertension and severe COVID-19 and hypertensive controls. (PDF)

S2 Table Correlations between the changes in plasma biomarkers and clinical parameters at day 4 and day 7. (PDF)

S3 Table Baseline characteristics and clinical outcomes of the 32 patients with severe COVID-19 according to dexamethasone use. (PDF)

S4 Table Serial analyses of clinical parameters and plasma biomarkers at days 1, 4, and 7 according to dexamethasone use. (PDF)

S1 Methods Calculation of area under the curve. (PDF)
