# Supplementary material for: Dexamethasone may improve severe COVID-19 via ameliorating endothelial injury and inflammation: A preliminary pilot study
Source: PLoS One. 2021 Jul 2;16(7):e0254167. doi: 10.1371/journal.pone.0254167 (PMC8253399; doi:10.1371/journal.pone.0254167)
Supplement: S1 Table — (PDF) [file pone.0254167.s001.pdf]

S1 Table. Comparing plasma biomarker concentrations between patients with hypertension and severe COVID-19 and hypertensive controls.

	Severe COVID-19 (n = 18)	Controls (n = 4)	*P*
Ang-2, pg/mL	1050 (730–1587)	1146 (1104–1196)	0.733
sTie2, pg/mL	16969 (13096–21040)	19689 (17898–21305)	0.268
Endocan, pg/mL	1048 (558–1819)	383 (337–458)	0.089
ICAM-1, pg/mL	413949 (293433–697463)	514049 (372715–623696)	0.798
IL-6, pg/mL	27.4 (11.9–38.6)	3.1 (2.6–3.7)	0.007
sRAGE, pg/mL	5320 (3581–7454)	1686 (1651–1979)	0.008
SP-D, pg/mL	5912 (2938–11080)	6269 (5423–6667)	0.932
Syndecan-1, pg/mL	5107 (4301–8511)	2851 (2282–3520)	0.008
TNF-α, pg/mL	7.1 (5.1–9.5)	7.0 (6.3–7.7)	0.798
vWF, pg/mL	4647 (2346–8798)	2723 (1674–3647)	0.148
IFN-α, pg/mL	136.9 (57.8–328.7)	14.9 (13.8–16.1)	0.002

Data are presented as median (interquartile range) and *P*-values were calculated using the Mann-Whitney *U* test.

Ang-2, angiopoietin-2; COVID-19, coronavirus disease 2019; ICAM-1, intercellular adhesion molecule-1; IFN-α, interferon-α; IL-6, interleukin-6; sTie2, soluble form of the Tie2 receptor; sRAGE, soluble receptor for advanced glycation end-products; SP-D, surfactant protein D; TNF-α, tumor necrosis factor-α; vWF, von Willebrand factor.
