# Supplementary material for: Dexamethasone may improve severe COVID-19 via ameliorating endothelial injury and inflammation: A preliminary pilot study
Source: PLoS One. 2021 Jul 2;16(7):e0254167. doi: 10.1371/journal.pone.0254167 (PMC8253399; doi:10.1371/journal.pone.0254167)
Supplement: S2 Table — (PDF) [file pone.0254167.s002.pdf]

S2 Table. Correlations between the changes in plasma biomarkers and clinical parameters at day 4 and day 7.

	Two-tailed Spearman correlation coefficient (non-parametric)									
**Δ Day 4**	Ang-2	sTie2	Endocan	ICAM-1	IL-6	sRAGE	SP-D	Syndecan-1	TNF-α	vWF
BT	0.18	0.15	0.29	0.15	0.33	-0.06	-0.53	0.24	0.39[a]	0.17
RR	0.48[b]	-0.05	0.12	-0.12	0.08	0.23	-0.19	0.12	0.07	-0.29
SpO_2/FiO_2	0.17	-0.12	-0.42	-0.10	-0.03	0.22	0.39[a]	-0.27	-0.22	-0.07
WBC	0.04	-0.13	0.29	-0.06	0.05	-0.24	-0.22	-0.19	0.21	0.17
LDH	0.003	-0.14	-0.04	0.06	0.09	0.47[b]	-0.39	-0.13	0.10	-0.17
CRP	0.61[b]	0.26	-0.01	0.41[a]	0.49[b]	0.41[a]	-0.14	-0.10	0.35	0.29
Rad score	0.42[a]	0.02	0.06	0.14	0.39[a]	0.46[b]	0.09	-0.15	0.30	-0.09
**Δ Day 7**	Ang-2	sTie2	Endocan	ICAM-1	IL-6	sRAGE	SP-D	Syndecan-1	TNF-α	vWF
BT	0.19	0.35	0.49[b]	0.26	0.37[a]	0.05	-0.67	0.23	0.34	0.11
RR	0.28	-0.29	0.01	0.03	-0.08	0.01	0.20	0.15	0.14	-0.37
SpO_2/FiO_2	0.41[a]	0.15	-0.38	0.11	0.09	0.27	0.44[a]	-0.01	0.13	0.26
WBC	0.18	-0.15	0.16	0.004	0.15	0.04	-0.08	-0.13	0.31	0.18
LDH	0.17	0.01	-0.06	0.14	0.35	0.48[b]	0.12	0.03	0.41[a]	0.13
CRP	0.64[b]	0.28	-0.04	0.41[a]	0.44[a]	0.42[a]	-0.08	0.02	0.32	0.16
Rad score	0.66[b]	0.30	-0.25	0.37[a]	0.45[a]	0.42[a]	0.18	-0.04	0.41[a]	0.20

Ang-2, angiopoietin-2; BT, body temperature; CRP, C-reactive protein; FiO_2, fraction of inspired oxygen; ICAM-1, intercellular adhesion molecule-1; IL-6, interleukin-6; LDH, lactate dehydrogenase; SpO_2, pulse oximetric saturation; Rad score, radiologic score; RR, respiratory rate; sTie2, soluble form of the Tie2 receptor; sRAGE, soluble receptor for advanced glycation end-products; SP-D, surfactant protein D; TNF-α, tumor necrosis factor-α; vWF, von Willebrand factor; WBC, white blood cell.

[a] $P < 0.05$.

[b] $P < 0.01$.
