# Supplementary material for: Dexamethasone may improve severe COVID-19 via ameliorating endothelial injury and inflammation: A preliminary pilot study
Source: PLoS One. 2021 Jul 2;16(7):e0254167. doi: 10.1371/journal.pone.0254167 (PMC8253399; doi:10.1371/journal.pone.0254167)
Supplement: S3 Table — (PDF) [file pone.0254167.s003.pdf]

S3 Table. Baseline characteristics and clinical outcomes of the 32 patients with severe COVID-19 according to dexamethasone use.

	Dexamethasone (n = 20)	No dexamethasone (n = 12)	*P*
**Baseline characteristics**			
Age, years	70 (59–75)	69 (60–79)	0.953
Male sex	11 (55)	6 (50)	0.784
Body mass index, kg/m^2	26.9 (23.4–31.0)	22.0 (20.7–24.0)	0.001
Smoking status			0.198
Ever smoker	7 (35)	7 (58)	
Never smoker	13 (65)	5 (42)	
Comorbidities			
Diabetes	8 (40)	0	0.014
Hypertension	12 (60)	6 (50)	0.581
Chronic lung disease	5 (25)	2 (17)	0.683
Liver cirrhosis	1 (5)	0	>0.99
Malignancy	1 (5)	0	>0.99
Charlson Comorbidity Index	3.0 (2.0–4.0)	3.0 (1.5–3.5)	0.460
Time from symptom onset to day 1, days	8 (7–10)	9 (4–11)	0.891
ARDS	5 (25)	2 (17)	0.683
qSOFA score	1 (0–1)	1 (0–1)	0.966
Type of oxygen support			<0.001
Mechanical ventilation	5 (25)	1 (8)	
High-flow nasal cannula	11 (55)	0	
Supplemental oxygen	4 (20)	11 (92)	
Remdesivir	11 (55)	0	0.002
Vital signs and laboratory data			
Body temperature, °C	37.8 (37.4–38.6)	38.0 (37.3–38.3)	0.640
Mean blood pressure, mmHg	84 (74–96)	80 (71–89)	0.391
Heart rate, beats/min	89 (76–95)	83 (79–88)	0.508
Respiratory rate, breaths/min	21 (20–24)	20 (20–24)	0.735
SpO_2/FiO_2	196 (172–272)	377 (291–408)	<0.001
Creatinine, mg/dL	0.7 (0.6–0.8)	0.8 (0.6–0.8)	0.654
White cell count, $1000/mm^3$	7.0 (4.2–8.3)	4.8 (3.4–5.8)	0.039
Total bilirubin, mg/dL	0.7 (0.5–1.0)	0.6 (0.5–0.8)	0.410
Lactate dehydrogenase, IU/L	408 (313–470)	357 (254–434)	0.259
C-reactive protein, mg/L	111 (65–183)	47 (18–92)	0.014
Glucose, mg/dL	141 (118–199)	116 (102–128)	0.037
Radiologic score	5 (3–6)	2 (1–4)	0.006
Cycle threshold value	25.1 (20.7–28.7)	25.9 (19.7–29.8)	0.815
**Clinical outcomes**			
Oxygen-free days at day 28	13.5 ± 8.9	13.3 ± 9.3	0.723
Length of hospital stay, days	21.9 ± 16.7	16.3 ± 4.3	0.666
Hospital mortality	1 (5)	0	>0.99
Superinfection	2 (10)	0	0.516

Data are presented as number (%), mean ± standard deviation, or median (interquartile range). The *P*-values were calculated using the Mann-Whitney *U* test for continuous variables and using the chi-squared test or Fisher's exact test for categorical variables.

ARDS, acute respiratory distress syndrome; COVID-19, coronavirus disease 2019; FiO_2, fraction of inspired oxygen; SpO_2, pulse oximetric saturation; qSOFA, quick Sequential Organ Failure Assessment.
