# Supplementary material for: Dexamethasone may improve severe COVID-19 via ameliorating endothelial injury and inflammation: A preliminary pilot study
Source: PLoS One. 2021 Jul 2;16(7):e0254167. doi: 10.1371/journal.pone.0254167 (PMC8253399; doi:10.1371/journal.pone.0254167)
Supplement: S4 Table — (PDF) [file pone.0254167.s004.pdf]

S4 Table. Serial analyses of clinical parameters and plasma biomarkers at days 1, 4, and 7 according to dexamethasone use.

	Day 1	Day 4	Day 7	AUC	*P*
**Clinical parameters**					
Body temperature, °C					0.161
DXM group	37.8 (37.4–38.6)	36.9 (36.7–37.5)	37.0 (36.8–37.4)	-0.6 (-1.2 to -0.2)	
Non-DXM group	38.0 (37.3–38.3)	37.4 (37.0–38.0)	37.2 (37.0–37.3)	-0.4 (-0.7 to -0.1)	
Respiratory rate, breaths/min					0.646
DXM group	21 (20–24)	20 (20–24)	20 (20–21)	0 (-3 to 0)	
Non-DXM group	20 (20–24)	20 (20–23)	20 (20–21)	0 (-1 to 0)	
SpO_2/FiO_2					0.052
DXM group	196 (172–272)	240 (207–272)	268 (234–338)	40 (-3 to 70)	
Non-DXM group	377 (291–408)	348 (272–404)	346 (291–379)	-2 (-32 to 28)	
Lactate dehydrogenase, IU/L					0.755
DXM group	408 (313–470)	335 (291–400)	325 (261–386)	-57 (-87 to 2)	
Non-DXM group	357 (254–434)	300 (283–331)	302 (243–350)	-36 (-87 to -3)	
C-reactive protein, mg/L					<0.001
DXM group	111 (65–183)	27 (19–51)	8 (4–24)	-58 (-113 to -30)	
Non-DXM group	47 (18–92)	43 (16–116)	45 (4–114)	10 (0 to 30)	
Glucose, mg/dL					0.284
DXM group	141 (118–199)	210 (115–274)	192 (111–248)	11 (-21 to 87)	
Non-DXM group	116 (102–128)	105 (99–116)	104 (98–144)	-5 (-14 to 11)	
Radiologic score					0.008
DXM group	5 (3–6)	3 (2–4)	2 (2–3)	-1 (-3 to 0)	
Non-DXM group	2 (1–4)	3 (2–3)	3 (2–4)	0 (0 to 1)	
Cycle threshold value					0.196
DXM group	25.1 (20.7–28.7)	27.7 (26.1–30.1)	29.5 (26.6–34.8)	3.9 (2.1 to 4.8)	
Non-DXM group	25.9 (19.7–29.8)	27.3 (26.0–33.6)	31.3 (26.9–34.8)	5.3 (3.7 to 5.8)	
**Plasma biomarkers**					
Ang-2, pg/mL					0.001
DXM group	1417 (978–2453)	969 (662–1757)	1100 (569–1362)	-294 (-680 to -140)	
Non-DXM group	929 (897–1155)	1174 (803–1281)	1145 (800–1958)	103 (-7 to 252)	
sTie2, pg/mL					0.006
DXM group	17464 (13901–22051)	14176 (11166–18068)	15002 (11668–19049)	-3044 (-4414 to -484)	
Non-DXM group	14278 (11835–16423)	13901 (11294–18637)	13029 (12294–17970)	90 (-1268 to 1595)	
Endocan, pg/mL					0.457
DXM group	821 (293–1564)	660 (337–885)	686 (340–1417)	12 (-583 to 277)	

Non-DXM group	1101 (697–1880)	676 (639–1881)	536 (373–901)	-167 (-420 to 14)	
ICAM-1, pg/mL					0.043
DXM group	448852 (347428–640257)	416283 (301071–576069)	420937 (309873–525985)	-39166 (-56899 to -18261)	
Non-DXM group	380511 (279395–560645)	339304 (279152–582766)	384361 (266984–524032)	1835 (-39825 to 19535)	
IL-6, pg/mL					0.107
DXM group	30.5 (5.1–49.6)	5.1 (3.6–7.9)	4.9 (2.1–9.2)	-20.3 (-33.0 to 0.17)	
Non-DXM group	17.7 (13.0–28.1)	10.1 (7.2–21.7)	5.2 (3.0–12.8)	-3.3 (-7.7 to 0.5)	
sRAGE, pg/mL					0.023
DXM group	6141 (3816–8206)	2514 (1789–4207)	1345 (1022–1863)	-2690 (-3986 to -1377)	
Non-DXM group	3740 (2345–6055)	3255 (2038–4670)	1619 (1389–2001)	-370 (-2179 to 301)	
SP-D, pg/mL					0.650
DXM group	7731 (2854–14272)	15018 (9566–24131)	15559 (8356–24267)	5308 (383 to 9774)	
Non-DXM group	5027 (3263–6379)	10744 (5782–14746)	12750 (6683–17433)	3209 (1083 to 6610)	
Syndecan-1, pg/mL					0.483
DXM group	5639 (4489–7849)	7478 (4371–9633)	7865 (5809–10756)	1164 (22 to 1829)	
Non-DXM group	4562 (3603–7734)	6411 (5006–9753)	7351 (4738–8829)	1543 (514 to 2129)	
TNF-α, pg/mL					0.010
DXM group	7.9 (6.3–9.8)	6.0 (5.2–8.1)	5.8 (5.2–7.6)	-1.3 (-2.7 to 0.1)	
Non-DXM group	7.9 (5.1–8.7)	6.8 (6.3–8.7)	6.3 (5.3–8.9)	0.5 (-0.6 to 1.5)	
vWF, pg/mL					0.107
DXM group	6805 (2885–8807)	3416 (2394–6585)	3264 (2181–7563)	-501 (-2870 to 344)	
Non-DXM group	2328 (1693–2884)	2526 (1755–3685)	4439 (1421–6150)	254 (-466 to 1108)	
IFN-α, pg/mL					0.836
DXM group	91.7 (41.3–161.6)	34.4 (28.1–41.5)	34.4 (24.1–39.0)	-47.5 (-92.8 to -3.3)	
Non-DXM group	148.5 (102.6–310.7)	53.9 (42.4–214.6)	41.6 (35.4–65.7)	-79.2 (-112.8 to -5.8)	

To avoid multiple comparisons, the median (interquartile range) values for the AUCs of continuous variables were calculated with repeated measurements. The *P*-values were calculated using the Mann-Whitney *U* test.

Ang-2, angiopoietin-2; AUC, area under the curve; DXM, dexamethasone; FiO_2, fraction of inspired oxygen; ICAM-1, intercellular adhesion molecule-1; IFN-α, interferon-α; IL-6, interleukin-6; SpO_2, pulse oximetric saturation; sTie2, soluble form of the Tie2 receptor; sRAGE, soluble receptor for advanced glycation end-products; SP-D, surfactant protein D; TNF-α, tumor necrosis factor-α; vWF, von Willebrand factor.
