# Supplementary material for: Dexamethasone may improve severe COVID-19 via ameliorating endothelial injury and inflammation: A preliminary pilot study
Source: PLoS One. 2021 Jul 2;16(7):e0254167. doi: 10.1371/journal.pone.0254167 (PMC8253399; doi:10.1371/journal.pone.0254167)
Supplement: S1 Methods — (PDF) [file pone.0254167.s005.pdf]

S1 Methods. Calculation of area under the curve.

The area under the curve (AUC) is calculated by adding the areas under the graph between each pair of consecutive observations. If we have measurements y_1 and y_2 at times t_1 and t_2, then the AUC between those two times is the product of the time difference and the average of the two measurements. Thus, we get $(t_2 - t_1)(y_1 + y_2)/2$. For this study, the AUC was calculated relative to baseline values (y_0) to standardize for potential baseline imbalance.

If we have n + 1 measurements y_i at times t_i (i = 0, …, n), then the AUC is calculated as:

$$\text{AUC}=\frac{1}{2}\sum_{i=0}^{n-1}(t_{i+1}-t_i)(y_i+y_{i+1}-2y_0).$$

Consider the data for one patient with severe COVID-19 in the present study. At days 1, 4, and 7, the Ang-2 concentrations are 3641.74, 1120.29, and 1359.25 pg/mL, respectively. Thus, we have

$$\text{AUC}=(4-1)\times\frac{(3641.74+1120.29-2\times3641.74)}{2}+(7-4)\times\frac{(1120.29+1359.25-2\times3641.74)}{2}$$

= -10988.09 pg day/mL.

If we standardize by the length of the study, 6 days, we get -10988.09/6 = -1831.35 pg/mL.
